# AGE AND EXERCISE DIFFERENTIALLY INFLUENCED MENTAL HEALTH DURING THE PANDEMIC

**DOI:** 10.1093/geroni/igad104.3118

**Published:** 2023-12-21

**Authors:** Zohra Feliachi, Julie Hicks Patrick

**Affiliations:** West Virginia University, Morgantown, West Virginia, United States; West Virginia University, Morgantown, West Virginia, United States

## Abstract

With over 103,436,829 confirmed COVID-19 cases and more than 1.13 million deaths in the USA between January 2020 and August 2023 (WHO, 2023), the pandemic has affected most aspects of life. Regarding emotional wellbeing, most research has shown that older adults fared better than their younger peers (e.g., Ebert et al., 2022), although explanatory mechanisms for this effect have not been examined fully. We sought to fill this gap by examining the influence of age and exercise before and after vaccines were developed for the COVID-19 pandemic in the USA. Using data from the Behavioral Risk Factor Surveillance System (BRFSS), we compared a model in which age and exercise were used to examine the number of poor mental health days experienced. We compared responses from 423367 adults from 2018 and 2019 with responses from 417964 adults interviewed in 2021 and 2022. Results of a 4 (year) by 4 (age group) by 2 (exercise) ANOVA revealed significant effects for for each factor. More importantly, these main effects and two 2-way interactions were moderated by the 3-way interaction, F (9, 841330) = 2.57, p=.006. Adults who exercised after the pandemic was underway reported fewer poor mental health days relative to those who did not exercise, especially among those younger than age 45 years. Given the ongoing challenges to emotional wellbeing, younger and middle-aged adults may need additional support to age well. Exercise may provide one avenue to greater wellbeing, particularly in the face of non-normative and history-graded events like pandemics.

